# Early intervention: Bridging the gap between practice and academia

**DOI:** 10.1186/1753-2000-3-23

**Published:** 2009-09-04

**Authors:** Jörg M Fegert, Ute Ziegenhain

**Affiliations:** 1 Department of Child and Adolescent Psychiatry/Psychotherapy, University Hospital Ulm, Steinhövelst 5, 89075 Ulm, Germany

## Editorial

Prevention and early intervention have increasingly become a focus of basic and applied research in child and adolescent psychiatry. In recent years, the emergent field of infant psychiatry has made significant progress. Many countries in the world try to invest more in prevention and intervention programs at the beginning of life, in an effort to decrease later health costs related to psychiatric disorders in childhood and adolescence [1]. Child and Adolescent Psychiatry and Mental Health provides an international forum for addressing important and timely issues in child mental health. In this context, we present a special section on early intervention in infants and preschoolers, in order to give an overview of the latest developments in this field and new research and practical reports from different settings and countries.

The first roots of early intervention can already be traced in Fröbel's kindergarten movement in the beginning of the 18th century. Much more recently, the best known and funded interventions include the Head Start programs that were initiated in the 1960s and have lasted until today. The Head Start provided a centralized service system that began with addressing the effects of poverty experienced in early life, and eventually was extended to other high-risk groups, including disabled and abused children [2]. Although Head Start originally focused on supporting intellectual development, the program eventually aimed at promoting the development of the child "as a whole" [3]. Associated with this perspective, the importance of an early start of intervention, and also the central role of parents in supporting or hindering positive development was emphasized [4-6]. In the last two decades this latter perspective was heavily informed by the attachment theory.

Actually, it was in the field of early and preventive intervention that attachment theory has proven its practical relevance to the largest extent. Intervention programs that are grounded in attachment theory and research and that focus explicitly on enhancing parental sensitive behavior and attachment security have been developed and applied to a great extent.

Although the evaluations of these attachment-based programs are in large part promising, especially with respect to enhancing parental sensitive behavior (while more modest success has been achieved in enhancing attachment security), there is discussion as to the optimal way of intervening.

For example one long standing discussion in the field is the discussion about the duration and the focus of interventions. In their narrative review of 15 attachment programs Egeland and his colleagues [7] concluded that long-term and frequent interventions with a more broader focus addressing both parental behavior and their representations should be considered most effective. In contrast, van IJzendoorn et al. [8,9] argued that short-term, and less broad interventions with a behavioral focus on parental sensitivity that only focus on sensitive maternal behavior appear to be more successful. The latter perspective was corroborated by a meta-analysis including 70 published intervention studies [9]. However, questions still remain about what interventions work for whom. Thus, practitioners argue that especially high-risk groups need to be administered more intensive and long term programs. The above mentioned meta-analysis included families at risk and special evaluation had be done for this subgroup. Nevertheless, newer and well evaluated intervention programs for high risk groups are more intensive and proved to be successful [10-12]. With respect to the specificity of risk factors or disorders, clearly further research is needed to find out which intervention components and program characteristics have to be tailored to fit for different families and clinical groups.

A central aim of intervention is to prevent or at least buffer negative developmental outcomes for children. To this end, early intervention is critical. Indeed, intervention programs such as the well evaluated and replicated nurse family partnership program [13] have impressively revealed the positive impact of a "healthy start to life" on maternal behavior and attitudes towards the child, or on the reduction of following pregnancies or the decrease of alcohol or drug consumption of the mothers [13-15]. The idea of the effectiveness of an early beginning of intervention is theoretically underpinned by the concept of a sensitive period of fast neuropsychological growth, and the influence of early social-emotional experiences, respectively attachment regulation processes as well as the ability to cope with stress. Moreover, in the case of extreme negative early attachment experiences such as maltreatment, it is supposed that even irreversible neurological damages can be caused [16-18]. However, currently the empirical evidence for a sensitive period is still rather thin. In any case there is a lack of evidence that would allow to draw conclusions about implications for the practice of intervention [19-21].

There is currently a growing interest in neurobiological, psycho physiological, and gene-environment interaction effects on developing attachment relationships, and, in particular, in disorganized and/or disordered attachment. This new directions are enhancing the traditional issues of attachment theory and research that were predominantly focused on behavioral regulation in parent-child interaction, and the influence of parental sensitivity on individual differences in the quality of attachment. They address the issue of the role of nonparental influences in developing attachment relationships. This is especially relevant for children in need of intervention, such as children with disorganized attachment behavior or children with disordered patterns of attachment, respectively reactive attachment disorder. These are maltreated or neglected children, and/or children in foster care, adopted children, and/or children who experienced early institutional deprivation as for example the Romanian children [22,23].

In fact, results from a previous meta-analysis on disorganized attachment revealed only a low correlation between disorganized attachment and parental sensitivity [24]. Thus, parental sensitivity, respectively childrearing antecedents alone cannot predict disorganized attachment. There is increasing evidence that some infants are more susceptible to stress exposure than others, and that such susceptibility is temperamentally and/or genetically based [22,23,25]. Thus, children may respond differently to inadequate or changing environments. In previous studies on psycho physiological measures significant cortisol increases in disorganized infants in a moderately stressful situation (the so called "strange situation)" were reported, suggesting vulnerability in coping with stress in these children [26]. In addition, it was suggested that neonatal temperamental effects may interact with caregiving risk [27]. More recent studies of disorganized attachment investigated the interaction between genes and quality of parental care. In these studies the dopamine D4 receptor was employed as a candidate gene for infant attachment behavior [28,29] or the serotonin transmitter polymorphism (5HTTLPR) [30]. The latter polymorphism is considered to be related to the regulation of fear and anxiety [31].

In one study [29,32] the interaction between critical, respectively disrupted maternal behavior and DRD4 polymorphism (7 repeat allele) predicted infant disorganization. In addition, a relation between critical maternal behavior and infant disorganization was found, but not between infant disorganization and DRD4 polymorphism. However, infant genotype significantly interacted with maternal disrupted behavior.

In another study [28] the interaction between critical maternal behavior and DRD4 polymorphism did not predict infant disorganization. Also no relation between the DRD4 polymorphism and infant disorganization was found. Again, there was also no main effect of critical maternal behavior on infant disorganization. However, the mothers' unresolved loss or trauma predicted infant disorganization, namely, when the infant carried the long allele of the DRD4 gene (7 repeat allele). Spangler and Zimmermann (2007) [30] found a relation between the short form of the serotonin transmitter polymorphism (5HTTLPR) and infant disorganization only when mothers were low in responsiveness, but not in infants whose mothers were highly responsive [33].

In sum, these findings, suggesting differential susceptibility to environmental influences such as parental childrearing behavior, may have implications for the tailoring of future intervention programs. Clearly, the lessons from genetic variations call for useful specific programs for the needs of "specific children" [34]. In that the findings are in accord with the overall conclusion on genetic research speaking of a more individualized medicine. However, again, to date the present findings are far from sufficient to imply for immediate translation into practical guidelines for early intervention or for implementation into intervention programs.

Altogether, there is a lack of systematic links between research and application to practice. Sigel (1998) [35] indicated an urgent need of "science practitioning", meaning systematically translating science and research findings into practice, or, on the other hand, defining research problems out of practical relevance.

The articles in this special issue are the result of a conference on early intervention, held in Stuttgart, Germany, about the above mentioned gap between research and practice. The conference was founded by the Landesstiftung foundation Baden-Württemberg, and was supported by the prime minister of the state of Baden-Württemberg. The aim of the interdisciplinary conference was to discuss the still unsatisfying state of translating research findings into applicable and effective practice programs. In his opening words the prime minister emphasized how absolutely essential it is to enhance a positive development of children early on, not only for the children's individual well-being but also because they are a crucial human resource for the economic development of a country.

Thomas O'Connor and Mary Spagnola review and discuss the literature about recent findings on early stress exposure and severe deprivation in the context of developing attachment relationships and risk mechanisms, and, moreover, with respect to implications for clinical practice and directions for future studies.

Judit Gervai reviews evidence about the gene-interaction effects on developing attachment relationships, displaying an overview over the current state of research, including the impressing work of her own study group.

Carlo Schuengel, Mirjam Oosterman and Paula Sterkenburg discuss the contribution of psychobiological theories and research with respect to conceptual prerequisites of attachment theory in relation to psychobiological aspects, with respect to findings about the specific psychophysiological reactions of foster children, and with respect to treatment of and intervention with children with disordered attachment.

The papers give an overview about both the current state of research in the field of early and preventive intervention as well as about the future perspectives in research and practical application in this field.
